# Cell cycle related long non-coding RNAs as the critical regulators of breast cancer progression and metastasis

**DOI:** 10.1186/s40659-022-00411-4

**Published:** 2023-01-03

**Authors:** Amir Sadra Zangouei, Malihe Zangoue, Negin Taghehchian, Alireza Zangooie, Hamid Reza Rahimi, Ehsan Saburi, Mahya Sadat Alavi, Meysam Moghbeli

**Affiliations:** 1 Student Research Committee, Faculty of Medicine, Mashhad University of Medical Sciences, Mashhad, Iran; 2 Department of Medical Genetics and Molecular Medicine, School of Medicine, Mashhad University of Medical Sciences, Mashhad, Iran; 3 Cellular and Molecular Research Center, Birjand University of Medical Sciences, Birjand, Iran; 4 Department of Anesthesiology, Faculty of Medicine, Birjand University of Medical Sciences, Birjand, Iran; 5 Medical Genetics Research Center, Mashhad University of Medical Sciences, Mashhad, Iran; 6 Student Research Committee, Birjand University of Medical Sciences, Birjand, Iran

**Keywords:** Breast cancer, LncRNAs, Cell cycle, Liquid biopsy, Diagnosis

## Abstract

Cell cycle is one of the main cellular mechanisms involved in tumor progression. Almost all of the active molecular pathways in tumor cells directly or indirectly target the cell cycle progression. Therefore, it is necessary to assess the molecular mechanisms involved in cell cycle regulation in tumor cells. Since, early diagnosis has pivotal role in better cancer management and treatment, it is required to introduce the non-invasive diagnostic markers. Long non-coding RNAs (LncRNAs) have higher stability in body fluids in comparison with mRNAs. Therefore, they can be used as efficient non-invasive markers for the early detection of breast cancer (BCa). In the present review we have summarized all of the reported lncRNAs involved in cell cycle regulation in BCa. It has been reported that lncRNAs mainly affect the cell cycle in G1/S transition through the CCND1/CDK4-6 complex. Present review paves the way of introducing the cell cycle related lncRNAs as efficient markers for the early detection of BCa.

## Background

Breast cancer (BCa) accounts for almost 31% of all malignancies diagnosed in women, and ranks as the most frequent and second leading cause of cancer-related mortality in females [1, 2]. An estimated 2,261,419 newly diagnosed BCa patients and 684,996 deaths were globally recorded in 2020 [3]. BCa can be histopathologically categorized into ductal, lobular, tubular, and papillary carcinomas [4]. BCa is a heterogeneous molecular malignancy that is classified into HER2 positive, luminal A, luminal B, and triple-negative breast cancer (TNBC) [5, 6]. There are several common treatment options for BCa including local excision, radical surgery, chemotherapy, endocrine therapy, and tissue-targeted therapies [7]. However, therapeutic resistance is a common problem in BCa cases in which almost half of initially responsive breast tumors develop chemo resistance via different mechanisms [8, 9]. Replicative immortality is a hallmark of cancer that results from deregulation of the cell cycle machinery. Cyclin-dependent kinases (CDKs) and cyclins are the main cell cycle regulators. Cell cycle–targeted therapies have been gaining attention as a rational option for the inhibition of tumor cell proliferation and apoptosis induction [10–12]. Screening modalities enable us to detect pre-cancerous lesions or cancers at earlier stages where treatment options show higher efficacies to prolong the patients’ survival rates [13, 14]. Liquid biopsy is defined as the process of obtaining and analyzing cancer-derived biomarkers through intact circulating tumor cells (CTCs), exosomes, and circulating tumor DNA/RNA in biofluids [15, 16]. CTCs are passively shed off from a tumor into the bloodstream which may function as seeds for further cancer metastasis [17–19]. Compared to conventional solid biopsy, liquid biopsy is considered a relatively non-invasive and sensitive technique that can be utilized for the early diagnosis of cancer, stratification of patients, prognosis, and disease monitoring [20–24]. Long noncoding RNAs (lncRNAs) are implicated in diverse cellular mechanisms including cell development, proliferation, apoptosis, and migration via transcriptional modulation of target genes [25, 26]. As regulatory RNAs, lncRNAs perform their biological functions as guides, scaffolds, or decoys to regulate the expression of various genes [27]. Circulating lncRNAs show high stability particularly when embedded in exosomes or apoptotic bodies in biofluids [28, 29]. LncRNA profiling in body fluids could be clearly reflective of their expression aberrancies in the originating tumor site [30–33]. Deregulation of cell cycle progression is one of the main molecular bases of BCa that can be affected by the lncRNAs. Therefore, in the present review we summarized all of the lncRNAs that affect the BCa progression via cell cycle regulation (Table 1). This review paves the way of introducing a panel of cell cycle specific lncRNAs for the early detection of BCa.

**Table 1:** all of the lncRNAs associated with cell cycle regulation in BCa

LncRNA	Target	Samples	Year	Refs.
TUG1	CCND1 and CDK4	58 NT MDA-MB-231, MDA-MB-453, MDA- MB-468, T-47D, MCF-7, ZR-75, and SK-BR-3, and MCF-10A cell lines	2017	Fan [38]
LINC01355	CCND1 and FOXO3	48 NT MCF-7, MDA-MB-231 cell lines	2019	Ai [42]
CASC9	miR‑195/497	17 NT MCF-10A, MDA-MB-231, MDA-MB-468, MCF7, and MDA-MB-415 cell lines	2019	Shao [43]
FOXC2-AS1	CCND1, Cyclin D2, and Cyclin D3	56 NT MCF-10A and BC cell lines MDA-MB-468, MDA-MB-231, MDA- MB-436, and MCF-7 cell lines	2019	Yang [44]
AP000439.3	CCND1	MCF7, ZR-75-1, and T47D cell lines	2017	Zhang [48]
MAFG-AS1	miR-339-5p	50 NT MCF-7, BT474, T47D, MCF-10A, MDA- MB-231 and MDA-MB-468 cell lines	2020	Feng [49]
CCAT2	CCND1, Cyclin E1, CDK4, p15, and EZH2	120 NT MCF-7, MDA-MB-231, and MCF10A cell lines	2017	Deng [50]
ITGB1	Cdc25C, Cyclin B1, and E-cadherin	20 NT* MDA-MB-231, MCF-7, T47D, ZR-75-30, and 1590, and HBL-100 cell lines	2017	Yan [56]
PRNCR1-2	pCHK2 and pAKT	HS-578 T, and MDA-MB-231 cell lines	2019	Pang [61]
Lnc712	HSP90, Cdc37, and CDK2	MCF-10A, MDA-MB-231, MCF-7, and MCF-7/ADM cell lines	2020	Cui [63]
DSCAM‐AS1	miR‐137	30 NT MCF7, T47D, SK‐BR‐3, and MDA‐MB‐31 cell lines	2019	Ma [64]
SNHG1	miR‑573	50 NT MCF10A, MCF7, T47D, MDA-MB-231, and MDA-MB-468 cell lines	2020	Xiong [69]
HOST2	Let-7b	30 NT MDA-MB-231, and MDA-MB-468 cell line	2019	Zhang [70]
ASncmtRNA	Cyclin B1, CCND1, CDK1, and CDK4	MDA-MB-231, MCF7, and ZR-75-1 (CRL-1500) cell lines	2019	Fitzpatrick [77]
LINC01089	CDK4 and CDK6	63 NT MDA-MB-231, BT- 549, SUM-159, MDA-MB-468, SK-BR-3, MCF-7, YCCB1,T47D, and MCF-10A cell lines	2019	Yuan [83]
LINC00668	CDK4, Bcl-2, p21, AKT, and mTOR	T47D, MCF7, MDA-MB-231, MDA- MB-436, and MCF-10A cell lines	2019	Qiu [88]
PTENP1	Cyclin A2 and CDK2	MCF7, and 293 T cell lines	2017	Chen [92]
GHET1	EGFR, c-Myc, PI3K, AKT, Cyclin D1, MMP-2, and MMP-9	30 NT MCF-7 cell line	2019	Han [93]
MIR100HG	p27	MDA-MB-231 and BT549 cell lines	2018	Wang [98]
LINC00993	p16INK4A, p14ARF, p53, and p21	MDA-MB-231 and BT-549 cell lines	2019	Guo [99]
LincIN	p21	MCF- 10A and -10F, BT- 20, HCC-1937, MCF-7, MDA-MB-231, SK-BR-3, T-47D, ZR-75-1, MDA-MB-231luc, MCF10ADCIS, and SUM225 cell lines	2017	Jiang [100]
RP1	p27	HEK-293 T, MCF-7, T47D, SKBR3, MDA-MB-231, BT549, HCC38, HCC1937, and MCF-10A cell lines	2019	Jia [101]
MIAT	miR-302, miR-150, and miR-29c	31 NT NCCIT, MCF7, SKBR-3, PC-3, LNCaP, A-172, U-87, SH-SY5Y, HT-29, SW48, SW480, SW1116, AGS, HELA, Hep G2, MDA-MB-231, and DPSC cell lines	2018	Alipoor [106]
RUSC1-AS1	KLF2 and CDKN1A	48 NT MDA-MB-231, MCF-7, BT549, and MCF-10A cell lines	2019	Hu [110]
PVT1	miR-1207-5p	50 NT T-47D, MDA-MB-231, BT-474,MCF-7, SKBR3, and 293 T, T- 47D, MDA-MB-231, BT-474, and MCF-10A cell lines	2017	Yan [115]
MALAT1	miR-124	MCF-7, Bcap-37 and MDAMB-435S, HCC1937, ZR-75-1, HS578T, and MDA-MB-231 cell lines	2016	Feng [118]
ERINA	E2F1 and RB1	MCF10A, BT-20, HCC1937, HS578T, MDA-MB-MB231, ZR-75-1, BT-474, MDA-MB-231, MCF7, T47D, and 293 T cell lines	2020	Fang [119]
H19	E2F6 and E2F1	MCF-7, T47D, BT20, and MDA-MB-231 cell lines	2005	Berteaux [120]
Lnc-CDC6	CDC6 and miR‐215	105 normal and 837 breast cancer tissues MCF‐10A, MCF‐10AT, MCF‐10CA1A, and MCF‐10CA1H, MDA‐MB‐231, MDA‐MB‐468, MCF‐7, MDA‐MB‐453, ZR‐75‐1, Hs578T, T47D, and SK‐BR‐3 cell lines	2019	Kong [124]
PANDAR	Bmi1 and p16	BT474, SK-BR3, MCF-7, T47D, and MDA-MB-231 cell lines	2016	Sang [127]
UCA1	EZH2, p21, PI3K, and AKT	10 NT MCF-7, T47D, LCC2, and LCC9 cell lines	2019	Li [136]

^*^Normal (N) and Tumor (T) tissues

### Cyclins and cyclin-dependent kinases (CDK)

**Fig. 1:**
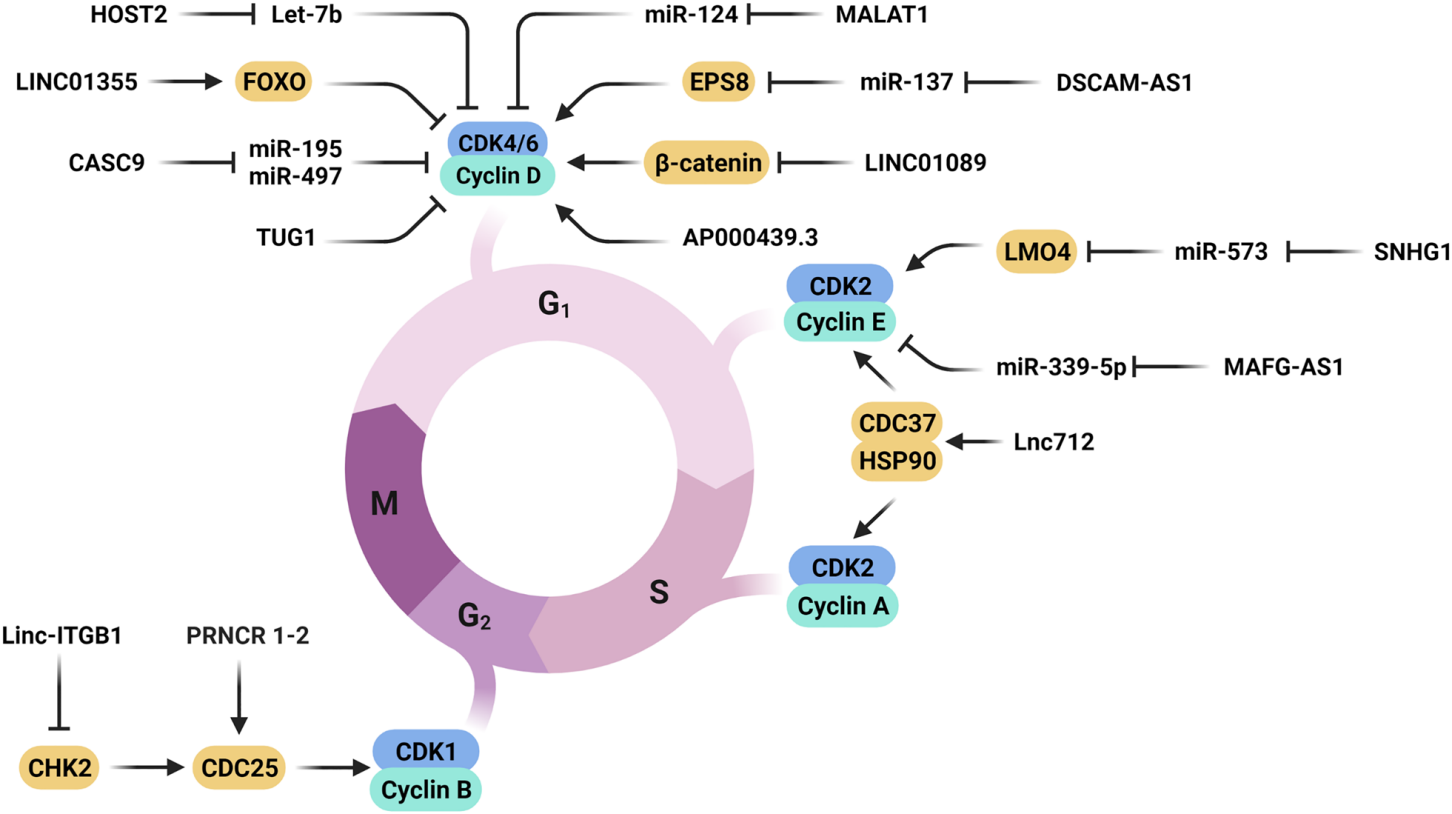
Role of lncRNAs in cell cycle regulation via Cyclin/CDK complexes in BCa. (Created with BioRender.com)

Cyclins and CDKs are the most important proteins involved in regulation of cell cycle progression [34]. Cyclin D1 (CCND1) and Cyclin-dependent kinase 4 (CDK4) have an important role in regulation of G1/S checkpoint. CCND1 proto-oncogene is a pivotal regulator of G1 to S phase progression via forming active complexes with CDK4 and CDK6 and retinoblastoma protein (RB) inhibition. It functions as a transcriptional co-regulator of different transcription factors and histone deacetylases [35, 36]. CDK6 phosphorylates members of the Rb protein family, leading to disengagement of the repressing complexes and releasing E2F transcription [37]. CDK4/6-CCND1 complex is one of the main targets of lncRNAs during tumor progression (Fig. 1). There was significant lncRNA TUG1 down regulation in BCa tissues compared with normal tissues that was correlated with lymph node metastasis and p53 mutation. TUG1 suppressed proliferation, invasion, and migration of breast tumor cells through G0/G1 arrest and promotion of apoptosis. However, the knockdown of TUG1 promoted BCa cell cycle progression via CCND1 and CDK4 up regulations [38]. Forkhead box O3 (FOXO3) belongs to the Forkhead family of transcription factors involved in BCa progression [39–41]. FOXO3 exerts its tumor suppressor role by CCND1 regulation [41]. There were significant lncRNA LINC01355 down regulations in BCa specimens and cell lines compared to normal margins and cell lines that was associated with larger tumor sizes and advanced clinical stages. LINC01355 promoted G0/G1 arrest through CCND1 down regulation. LINC01355 increased FOXO3 protein stability and induced the FOXO3 protein binding to the CCND1 promoter [42]. There were significant lncRNA Cancer Susceptibility 9 (CASC9) up regulations in both cell lines and BCa tissues. CASC9 promoted BCa cell proliferation and cell cycle progression, while suppressed cell apoptosis. CASC9 could act as an oncogene by miR-195/497 sponging. CASC9 up regulated the CCND1, CDK4, and BCL2, while down regulated the Caspase-3 (CASP3) [43]. It has been shown that there was lncRNA FOXC2-AS1 up regulation in BCa tissues compared with normal margins which was associated with poor prognosis. FOXC2-AS1 silencing induced G1 arrest and also promoted apoptosis in breast tumor cells [44].

Estrogen has pivotal roles in physiological and pathophysiological processes such as development of the normal mammary gland and tumorigenesis. Estrogen receptor (ER) is activated by estrogen and translocates into the nucleus to regulate gene expression through estrogen response elements (EREs). ER can also indirectly regulate target genes expression via interactions with other transcription factors such as AP1, SP1, and NF-κB. ER-regulated genes including c-Myc, CCND1, and BCL2 play crucial roles in cell proliferation and apoptosis [45]. Since, almost 70% of BCa cases are ER positive [46], Tamoxifen is the most frequently prescribed endocrine based treatment to block the effects of estrogen in BCa cells [47]. It has been reported that lncRNA AP000439.3 induced cell cycle progression in BCa cells via estrogen mediated CCDN1 up regulation [48]. The MAF bZIP transcription factor G antisense RNA 1 (MAFG-AS1) was significantly up regulated in BCa tissues compared with normal margins that was was correlated with worse prognosis and shorter survival. ER-positive breast tumors had generally higher MAFG-AS1 expression levels compared to ER-negative tumors. MAFG-AS1 up regulated the CDK2 via miR-339-5p sponging and promoted the G1/S phase transition [49]. There was significant lncRNA CCAT2 up regulation in BCa samples compared with normal margins which was correlated with advanced TNM stage, lymph node metastasis, and shorter survival. Knockdown of CCAT2 also reduced S phase cell proportion and suppressed cancer cell invasiveness [50].

Cyclin B1 plays a vital role as a regulatory subunit for CDK1 during G2 phase to mitosis progression. It also participates in checkpoint control, and its deregulation is observed in human cancers through its contribution in chromosomal instability. Cyclin B1 up regulation could serve as a signal to initiate the communication between tumor cells and their microenvironment [51, 52]. It can also be targeted by lncRNAs during the tumor progression (Fig. 1). The cell division cycle 25 (CDC25) family of phosphatase is a conserved eukaryotic protein family that includes CDC25A, CDC25B, and CDC25C isoforms. CDC25 phosphatases function as activators of the various CDKs [53]. Long intergenic non-coding RNA-integrin subunit β1 ITGB1 (linc-ITGB1) depletion down regulates the CDC25C and Cyclin B1 which are crucial for cell cycle progression [54, 55]. There were linc-ITGB1 up regulations in both BCa tissues and cell lines. Knockdown of linc-ITGB1 induced cell cycle arrest and significantly increased the cell number in the G0/G1 phase. The suppression of Cdc25C and cyclin B1 expressions via knockdown of linc-ITGB1 verified the role of linc-ITGB1 in cell cycle progression in BCa [56]. Checkpoint protein 2 (CHK2) is a ser/thr kinase which is required for DNA repair, cell cycle arrest, and apoptosis following DNA double-strand breaks (DSBs). It regulates cell cycle arrest by inhibition of CDC25 protein family of phosphatases that results in suppression of CDK-cyclin complexes. AKT is also a regulator of cell cycle progression, proliferation, and invasion that is activated by auto phosphorylation [57–60]. There was a significant prostate cancer-associated non-coding RNA (PRNCR1-2) up regulation in BCa tissues. CHK2 and AKT phosphorylation could be clearly affected by depletion of PRNCR1-2. PRNCR1-2 down regulation inhibited the cell cycle progression and invasion in BCa cells [61]. Co-chaperones such as Cdc37, p23, TAH1, HOP, and SGTA, are important regulatory proteins that assist heat shock protein 90 (HSP90) to exert its functions [62]. There was a correlation between lncRNA Lnc712 up regulation and breast tumor cell proliferation through HSP90/Cdc37/CDK2 axis. Knockdown of Lnc712 suppressed tumor cell growth via G0/G1 cell cycle arrest. Lnc712 regulated the cell cycle progression via CDK2 in which Lnc712 regulated CDK2 expression by inducing HSP90-CDC37 interaction and reinforcing HSP90-Cdc37 complex stability [63]. It was shown that DSCAM Antisense RNA 1 (DSCAM-AS1) was significantly overexpressed in Tamoxifen-resistant (TR) breast tumor samples. DSCAM-AS1 enhanced BCa cell proliferation, while suppressed apoptosis, proportion of cells in the G0/G1 phase, and was contributed to Tamoxifen resistance through miR-137 sponging and EPS8 up regulation. Following EPS8 down regulation, downstream genes c-Myc and CCND1 were also inhibited that suppressed cell cycle progression [64].

LIM domain only 4 (LMO4) belongs to the LIM-only sub-class of zinc finger proteins involved in development of mammary gland [65]. LMO4 induces cell cycle progression and facilitates cell proliferation by promoting CCND1 and cyclin E1 (CCNE1) expressions [66]. CCNE1 is essential for the pre-replication complexes. E-type cyclins also activate the CDK2 and Rb that subsequently activate E2F transcription factors. It facilitates the G1 to S phase transition and DNA synthesis by up regulation of histone proteins and cyclin A [67, 68]. CDK2-CCNE1 complex can also be regulated by lncRNAs during tumor progression (Fig. 1). It has been shown that there were Small Nucleolar RNA Host Gene 1 (SNHG1) up regulations in breast tumor tissues and cell lines. Suppression of SNHG1 expression inhibited the cell proliferation and migration, while induced G2/M arrest in breast tumor cells. SNHG1 sponged miR-573 to elevate the expression level of LMO4 in the breast tumor cells. SNHG1 knockdown also down regulated LMO4 and its target genes such as CCND1 and cyclin E1 expression [69]. It has been shown that there was significant lncRNA HOST up regulation in TNBC tumor tissues compared with normal breast epithelial cells. Silencing of HOST2 reduced cell proliferation and enriched G0/G1 cells in TNBC tumors. Down regulation of HOST2 increased let-7b expression as a tumor suppressor and decreased CDK6 expression [70].

Non-coding mitochondrial RNAs (ncmtRNAs) include sense (SncmtRNAs) and antisense (ASncmtRNAs) members [71–76]. SncmtRNAs are commonly expressed in all proliferating cells, demonstrating its putative function in cellular proliferation [71–73]. Suppression of AsncmtRNA expression is considered as a critical stage during tumorigenesis [12]. It has been reported that ASncmtRNA knockdown (ASK) promoted apoptosis of a variety of human tumor cell lines [75]. ASK inhibited cell proliferation through survivin, CDK1, CDK4, CCNB1, and CCND1 down regulations. MiR-4485-3p suppressed CCNB1 and CCND1 expressions in MDA-MB-231 cell line. ASK up regulated miR-5096 and miR-3609 that subsequently suppressed CDK1 [77].

### Signaling pathways

**Fig. 2:**
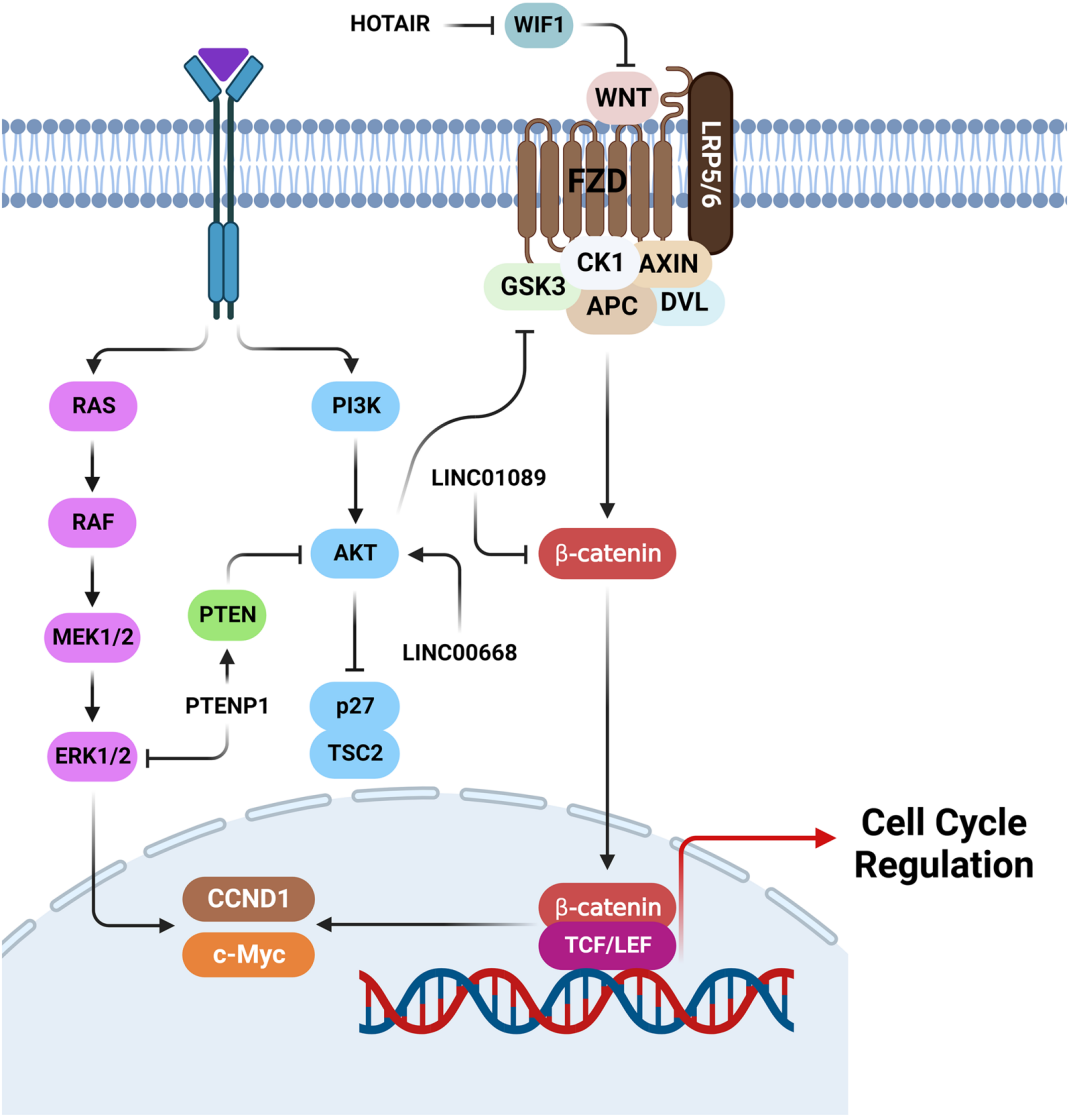
Role of lncRNAs in cell cycle regulation via WNT, PI3K/AKT, and MAPK signaling pathways in BCa. (Created with BioRender.com)

LncRNAs are involved in regulation of cell cycle progression in breast tumor cells through the regulation of WNT, PI3K/AKT, and MAPK signaling pathways (Fig. 2). The canonical Wnt/β-catenin signaling is a significant regulator of various biological processes, including cell proliferation, motility, and self-renewal [78]. In the absence of activating stimuli, destruction complex degrades the β-catenin through the ubiquitin–proteasome pathway [79]. The activity of degradation complex is suppressed upon stimulation by a Wnt signal. Subsequently, β-Catenin enters into the nucleus and binds to TCF/LEF transcription factor that modulates the WNT target genes [80]. In particular, abnormal accumulation of β-catenin has been observed in up to 50% of BCa patients that was significantly correlated with a poor prognosis [81, 82]. It has been observed that there was lncRNA LINC01089 down regulation in 80.9% of BCa cases. Deregulation of LINC01089 was markedly associated with age and lymph node metastasis in BCa patients. LINC01089 expression was negatively correlated with OS and DFS in BCa patients. LINC01089 inhibited breast tumor cell proliferation and invasion, while induced cell apoptosis and G0/G1 arrest by CCND1, CDK4, and CDK6 down regulations. LINC01089 decreased the levels of CCND1 and c-Myc expressions following the β-catenin down regulation [83].

CDK2 and CDK4 induce G1 phase to S phase transformation by modulating cell cycle-related proteins [84]. P21 serves as a tumor-suppressor gene that belongs to the family of CDK inhibitors. P21 negatively regulates CDK function, leading to cell division inhibition or promotion of apoptosis [85]. PI3K/AKT/mTOR axis has a pivotal role in cell cycle regulation [86]. It has been shown that the PI3K/AKT pathway can promote the G1/S transition through upregulation of Cyclin D via inhibiting the activity of GSK3-β and TSC2 and the expression of p27Kip1 [87]. LncRNA LINC00668 up regulation has been observed in BCa tissues compared with normal margins. LINC00668 expression was negatively correlated with prognosis of BCa patients. LINC00668 silencing significantly inhibited the cell cycle progression and colony formation, while promoted apoptosis in BCa cells. Knockdown of LINC00668 inhibited the AKT/mTOR pathway [88]. Phosphatase and tensin homolog (PTEN) is a tumor suppressor that functions as a negative regulator of PI3K/AKT pathway [89, 90]. The ancestral PTEN expression could be regulated by PTENp1 and thus influenced the carcinogenic process [91]. It has been reported that PTENp1 suppressed cell proliferation and motility through AKT signaling pathway, cyclin A2, and CDK2. PTENP1 reduced phosphorylation of Erk1/2 and p38 as important factors in MAPK signaling pathway. Therefore, PTENP1 regulated the BCa cell proliferation and migration by MAPK and AKT signaling pathways [92]. Following the inhibition of the PI3K/AKT pathway, cyclin D1 is down regulated that shows the background mechanism responsible for higher rates of apoptosis due to the increased proportion of G1 cells. HOTAIR along with polycomb repressive complex 2 alters PTEN, Wnt inhibitory factor 1 (WIF1), and p21 expression levels through regulation of histone H3 at the lysine 27th trimethylation. LncRNA GHET1 down regulation inhibited proliferative, invasive, and migratory potentials of cancer cells, while induced apoptotic cell death via increasing the proportion of cells in G1. GHET1 and c-Myc down regulations inhibited the PI3K/AKT axis in vitro and in vivo. GHET1 knockdown suppressed migratory and invasive capabilities of breast tumor cells via MMP-2 and MMP-9 down regulations [93].

### Cyclin-dependent kinase inhibitors

**Fig. 3:**
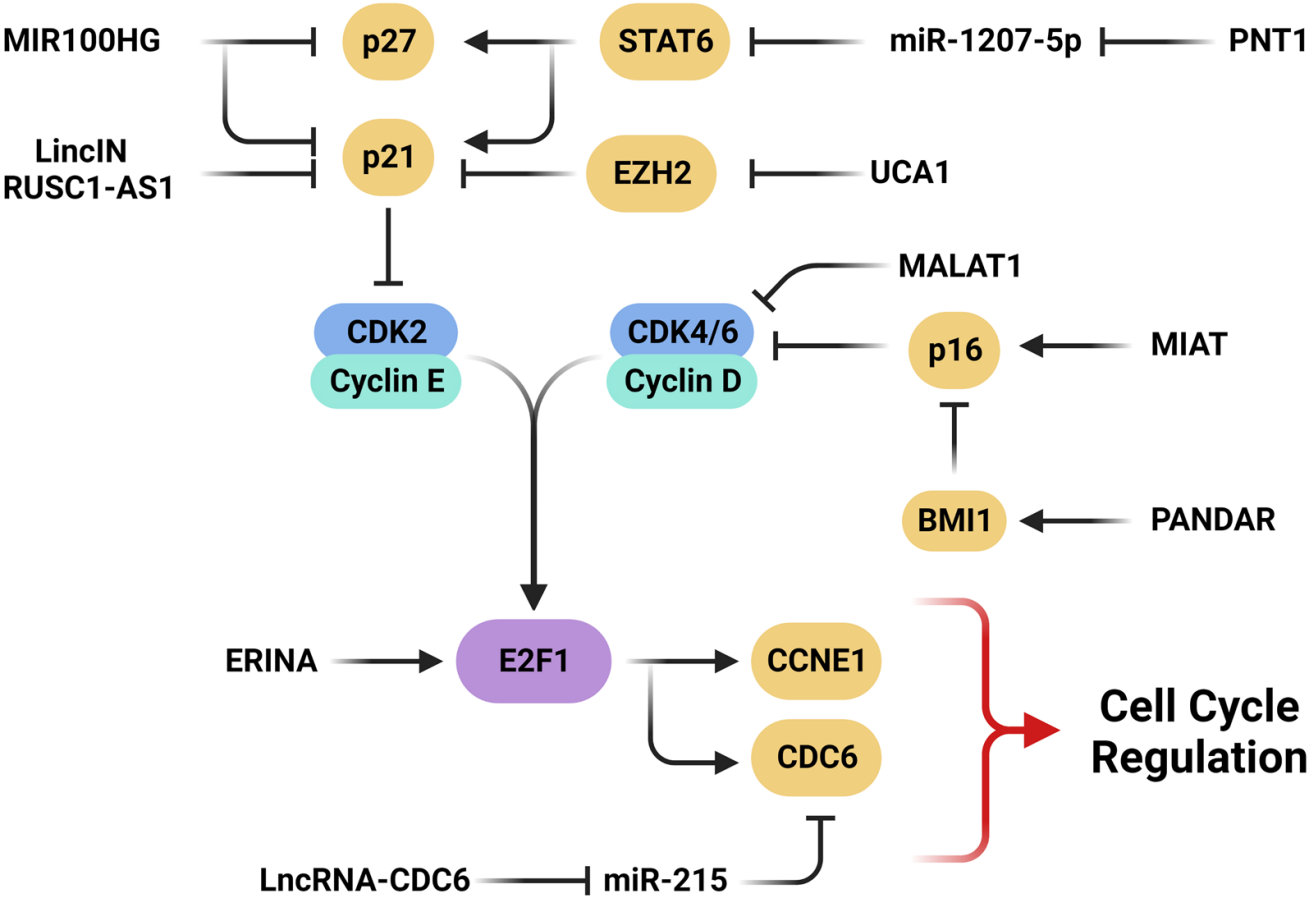
Role of lncRNAs in cell cycle regulation via CDK inhibitors and transcription factors in BCa. (Created with BioRender.com)

P16^Ink4a^ belongs to the INK4-class of cell-cycle inhibitors, which is involved in modulation of the cell cycle progression through inhibiting the S phase. P16 inhibits phosphorylation of RB family members via inhibiting cyclin D–CDK4/6 complex formation. Subsequently, p16 expression maintains the RB members in a hypo phosphorylated state, leading to G1 cell cycle arrest through the promotion of binding to E2F1 (Fig. 3). P16 expression is primarily regulated by transcriptional control and is necessary for tissue homeostasis, tumor suppression, and aging [94, 95]. P21 (p21^WAF1/Cip1^) is one of the most critical factors that induces cell cycle arrest in response to different intra-extracellular stimulations through both p53-dependent and p53-independent mechanisms. The nuclear localization of p21 is correlated with its inhibitory effect on cell cycle progression. It mediates numerous biological processes via inhibiting the CDK2 and CDK1, resulting in growth and cell cycle arrest at specific stages [96, 97]. CCND1 promotes the G1/S transition, while p21 and p27 inhibit cell cycle progression in the G1 phase via functioning on CDK4/6-cyclin D1 complex. It has been shown that there was significant lncRNA MIR100HG up regulation in TNBC tissues compared with other BCa subtypes. MIR100HG up regulation was also correlated with p21 and p27 down regulations, while CCND1 up regulation. MIR100HG enhanced the proliferation of TNBC cells through forming a triplex structure with p27 [98]. It has been shown that there was significant lncRNA LINC00993 down regulation in TNBC tissues. LINC00993 hindered cancer cell proliferation through inducing G0/G1 cell cycle arrest. It also regulated the translocation of E2F via two putative pathways: p14ARF- p53- p21-CDK2- Rb/E2, or p16INK4A-CDK4/6- Rb/E2 [99]. LncRNA LincIN up regulation was commonly observed in most of advanced BCa cases and is associated with a poorer prognosis. Through its interaction with the RNA-binding protein NF90, LincIN functions in translational regulation of p21 protein expression. Knockdown of LincIN up regulated the p21 [100]. There was significant RP1 up regulation in BCa that was correlated with a worse prognosis and poorer clinical parameters. RP1 promoted BCa cell growth and metastasis. P27kip1 suppressed BCa metastasis via reduction of Snail1. RP1 expression attenuated p27kip1 translation through direct interaction of RP1 with the p-4E-BP1/eIF4E complex. A significant association was observed considering the expression levels of Kruppel Like Factor 5 (KLF5) and Retinitis Pigmentosa 1 (RP1) in breast tumor cells. The p300 recruitment to the RP1 promoter by KLF5 was crucial for RP1 up regulation in BCa [101]. Myocardial infarction associated transcript (MIAT) is an lncRNA involved in various human disorders including myocardial infarction [102, 103], lung adenocarcinoma [104], and neuroendocrine prostate cancer [105]. There was MIAT up regulation in BCa samples. MIAT expression was markedly higher in ER- and PR-positive and p53 negative BCa samples as compared to ER- and PR-negative and p53 positive ones, respectively. High-grade ductal BCa also showed significant MIAT up regulation compared with low-grade tumors. It was concluded that MIAT promotes G1 arrest and cellular senescence through p16^Ink4A^ overexpression followed by CCND1 down regulation. MIAT was introduced as a therapeutic target which traps cancer cells in stable cell cycle arrest and can be used for suppressing cancer growth and triggering cellular senescence [106].

The cell cycle inhibitor p21 (CDKN1A) is involved in DNA damage response through different processes, including direct inhibition of DNA replication, and promoting cell cycle arrest by targeting P53 activity and regulating vital processes such as apoptosis, cell motility, and transcription. The primary function of CDKN1A is cell cycle arrest via inhibiting the activity of CDKs. Several studies have also indicated that CDKN1A may directly participate in DNA repair through the number of proteins involved in these processes. CDKN1A regulates the cell cycle progression at G1 phase through the inhibition of CDK2 or CDK4 complexes [107, 108]. Lysine-specific histone demethylase 1A (LSD1) is an amine oxidases which functions in the demethylation of mono- and di-methylated lysines [109]. There were significant RUSC1 Antisense RNA 1 (RUSC1-AS1) up regulations in BCa tissues and cell lines compared with controls. Expression level of RUSC1-AS1 was also directly correlated with tumor size and clinical grade; however, an inverse correlation was established considering RUSC1-AS1 levels and overall survival of BCa patients. RUSC1-AS1 knockdown suppressed proliferative, invasive, and migratory potentials of BCa cells. It also induced G0/G1 arrest, reduced cancer cell viability, and triggered their apoptosis. RUSC1-AS1 enhanced BCa growth through epigenetic silencing of KLF2 and CDKN1A [110]. CDKN1B, or p27 (KIP1), is a CDK inhibitor that inhibits cell cycle progression in the G0/G1 phase through different molecular mechanisms. CDKN1B also modulates cell motility and apoptosis. Cytoplasmic p27 plays a fundamental role in cell motility and migration by binding to RhoA and regulating the RhoA/ROCK cascade [111]. STAT family of transcription factors function in various aspects of cellular biology and are activated by different cytokines, growth factors, and hormones [112]. Following their activation, STATs dissociate from the receptor and translocate to the nucleus, where promotes the transcription of their target genes [113, 114]. It has been reported that lncRNA PVT1-derived miR-1207-5p induced colony formation and cell proliferation via STAT6 targeting in BCa. MiR-1207-5p also regulated cell cycle through suppression of CDKN1A and CDKN1B [115].

### Transcription factors and polycomb repressive complexes

Transcription factors and polycomb repressive complexes are the pivotal regulators of cell cycle checkpoints and components that can also be targeted by the lncRNAs during breast tumor progression (Fig. 3). E2F1 transcription factor is one of the critical cell cycle regulators that regulates DNA repair, apoptosis, and G1/S transition. E2F1 was identified as a tumor suppressor that can bind with pRB. The activity of E2F1 is reliant on its binding partners, including pRB (RB1), p107 (RBL1), and p130 (RBL2). Interaction of E2F1–pRB could suppress the regulation of target genes [116, 117]. It was reported that there was significant lncRNA MALAT1 up regulation in BCa tissues compared with normal margins. Silencing of MALAT1 inhibited breast tumor cell proliferation. MALAT1 increased breast tumor cell proliferation by miR-124 sponging and activating the CDK4/E2F1 axis [118]. LncRNA ERINA up regulation was found to be significantly associated with sensitivity to CDK inhibitors and poor survival of BCa cell lines and ER-positive BCa patients, respectively. ERINA increased drug resistance by targeting and sequestering the E2F1/RB1 [119]. It has been shown that E2F regulated the cell cycle progression via H19 in which H19 gene expression was suppressed by two E2F-dependent transcription inhibitors, including pRb and E2F6. H19 also enhanced the G1-S transition via binding to the E2F1 in breast tumor cells [120]. Cell division cycle 6 (CDC6) is a critical factor for the initiation and regulation of DNA replication. CDC6 expression is strictly regulated via E2F family of transcription factors [121]. It has been observed that suppression of CDC6 expression prompts G1/S phase arrest in the head and neck squamous cell carcinoma [122]. Moreover, CDC6 up regulation repressed the E-cadherin transcription in cervical cancer [123]. LncRNA–CDC6 regulated the BCa cell proliferation and metastasis through the miR‐215/CDC6 axis. It was also found that knockdown of lncRNA–CDC6 suppressed breast tumor cell proliferation via induction of G1 arrest. Moreover, there was a direct correlation between the lncRNA–CDC6 expression levels and higher tumor stages in BCa [124].

Bmi1 belongs to the polycomb group (PcG) protein family involved in cell cycle regulation mediated by p16^INK4A^ [125]. p16^INK4A^ causes cell cycle arrest and RB inhibition through blockage of CCND-CDK4/6 complex [126]. There were significant Promoter of CDKN1A Antisense DNA Damage Activated RNA (PANDAR) up regulations in BCa tissues and cell lines compared with normal counterparts. Silencing of PANDAR decreased cell growth and colony formation, while promoted G1/S arrest in breast cancer cells. Moreover, p16^INK4A^ was identified as a critical downstream target of PANDAR involved in the G1/S transition of BCa cells. PANDAR enhanced the binding of Bmi1 complex to the p16^INK4A^ promoter and promoted cell growth through the regulation of the PANDAR/Bmi1/p16^INK4A^ axis. PANDAR may also functions as a tumor-promoting factor and regulates the cell cycle of BCa cells by p16^INK4A^ down regulation [127]. About 70% of the BCa patients are estrogen receptor-positive (ER +) [128]. As an estrogen antagonist, tamoxifen is the most widely used endocrine therapy in BCa [129, 130]. In spite of the effectiveness of tamoxifen therapy for most of patients with ER + BCa, many tumors finally recur due to tamoxifen resistance [131, 132]. Deregulation of histone deacetylase, loss of ERα, and induction of unusual levels of estradiol are the mechanisms of tamoxifen resistance [133–135]. As a histone methyltransferase, zeste homolog 2 (EZH2) promotes the trimethylation of H3K27me3 in target genes. It has been reported that there was UCA1 up regulation in tamoxifen-resistant compared with tamoxifen-sensitive breast cancer. Knockdown of UCA1 resulted in G2/M arrest and alteration in CCND1 and p21 expressions. p21 was down regulated by EZH2 through H3K27me3 that was mediated by UCA1 [136].

## Conclusions

In the present review, we summarized all of the studies about the role of lncRNAs in cell cycle regulation in breast tumor cells. It has been reported that lncRNAs mainly affect the cell cycle in G1/S transition through the CCND1/CDK4-6 complex or CDK inhibitors. Due to the stability of lncRNAs in body fluids and the importance of the cell cycle during BCa progression, lncRNAs can be introduced as efficient non-invasive markers for the early detection of BCa tumors. Present review paves the way of introducing a non-invasive diagnostic panel marker by the cell cycle-associated lncRNAs in BCa.

## Data Availability

The datasets used and/or analyzed during the current study are available from the corresponding author on reasonable request.

## Abbreviations

LncRNAs: Long non-coding RNAs
BCa: Breast cancer
TNBC: Triple-negative breast cancer
CDKs: Cyclin-dependent kinases
CTCs: Circulating tumor cells
RB: Retinoblastoma protein
FOXO3: Forkhead box O3
ER: Estrogen receptor
EREs: Estrogen response elements
CDC25: Cell division cycle 25
CHK2: Checkpoint protein 2
DSBs: Double-strand breaks
HSP90: Heat shock protein 90
LMO4: LIM domain only 4
CCNE1: Cyclin-E1
ncmtRNAs: Non-coding mitochondrial RNAs
SncmtRNAs: Sense Non-coding mitochondrial RNAs
ASncmtRNAs: Antisense Non-coding mitochondrial RNAs
ASK: ASncmtRNA knockdown
MIAT: Myocardial infarction associated transcript
LSD1: Lysine-specific histone demethylase 1A
PcG: Polycomb group
CCND1: Cyclin D1
CDK4: Cyclin-dependent kinase 4
CASC9: Cancer Susceptibility 9
CASP3: Caspase-3
MAFG-AS1: MAF bZIP transcription factor G antisense RNA 1
Linc-ITGB1: Long intergenic non-coding RNA-integrin subunit β1 ITGB1
PRNCR1-2: Prostate cancer-associated non-coding RNA
DSCAM-AS1: DSCAM Antisense RNA 1
SNHG1: Small nucleolar RNA host gene 1
PTEN: Phosphatase and tensin homolog
KLF5: Kruppel like factor 5
RP1: Retinitis pigmentosa 1
RUSC1-AS1: RUSC1 Antisense RNA 1
PANDAR: Promoter of CDKN1A antisense DNA damage activated RNA
